# AI frontiers in emergency care: the next evolution of nursing interventions

**DOI:** 10.3389/fpubh.2024.1439412

**Published:** 2024-12-11

**Authors:** Zakaria Mani, Bander Albagawi

**Affiliations:** ^1^Nursing Department, Jazan University, Jazan, Saudi Arabia; ^2^Medical Surgical Department, College of Nursing, Imam Mohammad Ibn Saud Islamic University, Riyadh, Saudi Arabia; ^3^Medical Surgical Department, College of Nursing, University of Hail, Hail, Saudi Arabia

**Keywords:** artificial intelligence, machine learning, emergency nursing, emergency department, patient monitoring, clinical decision support, ethical considerations

## Abstract

This scoping review explores the utilization of artificial intelligence in emergency nursing, assessing its impact, potential benefits, and the obstacles faced in its adoption. It covers the scope of AI from advanced triage protocols to continuous monitoring of patients, assistance in diagnosis, and providing support for clinical decisions. The review notes that AI in emergency healthcare can lead to more efficient care and timely, data-driven actions, but also highlights significant issues such as safeguarding patient data, the necessity for dependable infrastructure, and concerns over discriminatory algorithms. The promise of AI in improving emergency healthcare practices and patient care is clear, yet the identified challenges must be carefully navigated to promote safe and ethical use. Further empirical research is called for to confirm the effectiveness of AI applications in the dynamic environment of emergency care setups.

## Introduction

The integration of Artificial Intelligence in emergency nursing has the potential to significantly transform patient care by improving outcomes, increasing workflow efficiency, and enhancing decision support systems (1). Despite the growing investment in and adoption of artificial intelligence in medicine, the applications of AI in an emergency setting remain unclear (2).

Emergencies present unique challenges and require quick and accurate decision-making (2).

The field of emergency nursing plays a crucial role in providing immediate and effective care to patients in the emergency department. With the advancement of technology, the integration of artificial intelligence in emergency nursing has the potential to revolutionize patient care delivery in the emergency department (1, 3). The future of AI in emergency nursing looks promising (4).

The integration of AI in emergency nursing holds promise for enhancing diagnostic capabilities. Machine learning algorithms can analyze vast amounts of data to assist in the early detection of critical conditions, leading to more timely and accurate diagnoses (5, 6). This can ultimately contribute to better patient outcomes and reduced treatment times in the emergency department (5). By analyzing patient data, medical literature, and treatment guidelines, AI systems can generate tailored treatment plans that take into account the individual patient’s characteristics and medical history (3, 6). AI has implications for treatment planning and decision support in emergency nursing. By analyzing patient data and leveraging evidence-based guidelines, AI systems can provide healthcare providers with personalized treatment recommendations, aiding in the delivery of tailored care to individual patients (1, 6).

The future of AI in emergency nursing not only holds promise for enhancing patient care but also poses potential challenges and ethical considerations that must be carefully addressed (1). As the integration of AI continues to evolve in emergency nursing, it is essential for healthcare providers to stay informed and prepared for the transformative impact of this technology on emergency care delivery. This scoping review aims to explore the current landscape of AI applications in emergency nursing and identify potential areas for future development and implementation. By understanding the possibilities and challenges associated with AI in emergency nursing, healthcare providers can better prepare for the changes that lie ahead and ensure the delivery of high-quality, efficient care to patients in critical situations.

### Method

A scoping review was conducted followed the Preferred Reporting Items for Systematic reviews and Meta-Analyses extension for Scoping Reviews (PRISMA-ScR) (7). A systematic and comprehensive search of electronic databases was conducted to identify relevant literature on the topic of artificial intelligence applications in emergency nursing.

The databases included PubMed, Scopus, Embase, and CINAHL. A search strings for each database was used considerably in this scoping review. To ensure a comprehensive and methodologically rigorous scoping review of artificial intelligence in emergency nursing, we employed a systematic approach to keyword search string development and refinement. We began by identifying the core concepts underpinning our research question: “artificial intelligence” and “emergency nursing.” Recognizing the breadth of terminology used within these domains, we brainstormed an extensive list of synonyms and related terms. This included terms like “machine learning,” “deep learning,” and “natural language processing” for AI, and “emergency department,” “emergency care,” “acute care,” and “critical care” for emergency nursing.

To capture relevant literature across various databases, we utilized a combination of keywords and database-specific features. Boolean operators were strategically employed to combine search terms and refine results. For instance, we used “AND” to connect concepts that must both be present, such as “artificial intelligence” AND “emergency nursing.” We used “OR” to broaden the search to include any of the listed synonyms, such as “triage” OR “prioritization.”

OR “patient assessment.”

Furthermore, we took advantage of controlled vocabularies offered by different databases. In PubMed, we incorporated relevant MeSH terms to ensure consistency with standardized medical terminology. Similarly, we utilized subject headings and other database-specific features to maximize the comprehensiveness of our search across other databases. Throughout the search process, we iteratively tested and refined our search strings based on the initial results. This involved adding more specific terms to narrow the scope when necessary and incorporating broader terms or synonyms when the initial search was too restrictive. This detailed record ensures transparency and allows for the replication of our search strategy by other researchers.

The search was further refined by including only peer-reviewed journal articles published in English. The time frame for the search was not restricted to capture the entire scope of the literature available to date 2024. Additional records were identified through hand-searching reference lists of included articles that meet the inclusion criteria of this study.

Inclusion criteria were set to identify studies that specifically focused on the application of AI in emergency nursing practices or were pertinent to the emergency department setting. The criteria were as follows:

1. Peer-reviewed journal articles discussing the use of AI and machine learning in emergency nursing.
2. Research focused on AI applications including triage, patient monitoring, diagnosis, treatment recommendations, and decision support within emergency departments. The five application areas within emergency nursing – triage, patient monitoring, diagnosis, treatment recommendations, and decision support – were selected based on a focused review of existing research activity within emergency departments, signifying their potential to transform emergency nursing practices.
3. Case studies, pilot projects, and empirical research highlighting the outcomes and effectiveness of AI interventions in emergency nursing.
4. Review articles summarizing the current state of AI technologies in emergency nursing, providing insights into future developments.

Excluded were editorial articles, commentaries, articles not in English, and those that did not specifically address AI in the context of emergency nursing.

**Figure 1:**
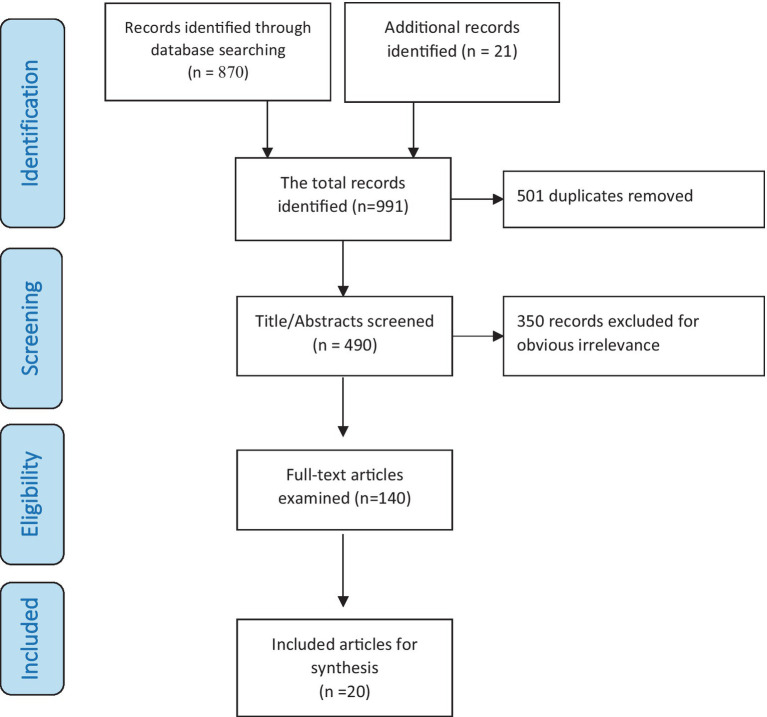
Flow diagram of search results.

To maintain the integrity of the search findings, Covidence Review software was utilized to initially to screen the titles and abstracts of publications. This preliminary filter was carried out by the two researchers, who then proceeded to a thorough full-text analysis of the articles that passed the initial screen. The process of collating information from papers that met the criteria was executed by researchers using Google Sheets and later cross-verified by either ZM or BA. Any differences in data interpretation were settled through further discussions. The selection process of the research results was illustrated in a PRISMA diagram (see Figure 1).

### Data extraction and analysis

The titles and abstracts of identified articles were screened for relevance to the review’s topics, and full texts were obtained for all articles that met the inclusion criteria. A standardized data extraction form was used to capture key information from each article, including objectives, AI technologies explored, study population, methods, main findings, and noted implications for clinical practice. Both quantitative and qualitative information was collected to provide a comprehensive overview of the field. Recurring patterns and narratives were identified through a thematic analysis of the data. The results were analyzed and synthesized according to AI application areas within emergency nursing: triage, patient monitoring, diagnosis, treatment planning, and decision support. Themes regarding the challenges, opportunities, and future prospects of AI in emergency nursing were also identified.

### Characteristics of the selected articles

This scoping review identified 20 studies exploring the intersection of AI and emergency nursing. This scoping review draws upon a diverse body of literature, as evidenced by the 20 selected articles published between 2019 and 2024. This timeframe highlights a recent surge in scholarly interest surrounding AI applications within emergency care. The methodological approaches employed vary considerably, reflecting the exploratory nature of the field. A significant portion of the articles (*N* = 10) are reviews, with systematic reviews (*N* = 5) further emphasizing the consolidation of existing knowledge. Case studies (*N* = 2) and retrospective analyses (*N* = 2) provide insights into real-world implementations and outcomes. Additionally, one study employed a machine learning and data mining approach for disease diagnosis.

Sample sizes for studies conducting primary research were diverse, ranging from a single case scenario to over 9 million emergency department visits. This range underscores the breadth of AI applications being explored, from individual patient care to system-wide interventions. Geographically, the research originated from various countries, including Korea, Italy, Taiwan, and China and reviews from various countries, indicating a global interest in leveraging AI to improve nursing emergency care. The variety in publication years, study designs, sample sizes, and geographical locations underscores the dynamism and global relevance of AI in emergency nursing (see Table 1).

**Table 1:** Characteristics of the selected articles.

No	Title	Year	Authors	Design	Sample size	Country
1	Reliability of ChatGPT for performing triage task in the emergency department using the Korean Triage and Acuity Scale	2024	Kim JH, Kim SK, Choi J, Lee Y.	Case study	202 clinical case scenarios	Korea
2	Artificial intelligence may enhance emergency triage and management	2023	Craca M, Coccolini F, Bignami E.	Case study	One case scenario	Italy
3	Application of Artificial Intelligence-Based Technologies in the Healthcare Industry: Opportunities and Challenges	2021	Lee D, Yoon SN	A review		
4	Artificial intelligence for clinical decision support for monitoring patients in cardiovascular ICUs	2023	Moazemi S, Vahdati S, Li J, et al	A systematic review		
5	Development and Validation of an Artificial Intelligence Electrocardiogram Recommendation System in the Emergency Department	2022	Tsai DJ, Tsai SH, Chiang HH, Lee CC, Chen SJ	A retrospective study	From August 2017 to November 2020, a total of 354,576 patient visits in the ED triage registry	Taiwan
6	Role of Artificial Intelligence in Patient Safety Outcomes: Systematic Literature	2020	Choudhury A, Asan O	A systematic review		
7	Remote patient monitoring using artificial intelligence: Current state, applications, and challenges.	2023	Shaik, T., Tao, X., Higgins, N., Li, L., Gururajan, R., Zhou, X., & Acharya, U R.	A review		
8	Artificial intelligence algorithm to predict the need for critical care in prehospital emergency medical services.	2020	Kang D, Cho K, Kwon O, Kwon J, Jeon K, Park SY, Lee Y, Park J, Oh B.	A retrospective observation cohort study	Total of 9,304,887 ED visits to 151 hospitals	Korea
9	Applications of Machine Learning Approaches in Emergency Medicine	2019	Shafaf N, Malek H.	A review		
10	Artificial Intelligence in Predicting Cardiac Arrest	2021	Alamgir A, Mousa O, Shah Z.	Scoping Review		
11	Artificial intelligence in disease diagnosis: a systematic literature review, synthesizing framework and future research agenda	2023	Kumar Y, Koul A, Singla R, Ijaz MF	A systematic review		
12	Machine Learning Versus Usual Care for Diagnostic and Prognostic Prediction in the Emergency Department: A Systematic Review.	2021	Kareemi H, Vaillancourt C, Rosenberg H, Fournier K, Yadav K.	A Systematic Review		
13	A comparison of deep learning performance against health-care professionals in detecting diseases from medical imaging: a systematic review and meta-analysis	2019	Liu X, Faes L, Kale AU, et al	A systematic review and meta-analysis		
14	Artificial Intelligence-Based Neural Network for the Diagnosis of Diabetes: Model Development.	2020	Liu Y.	Macnine learning and data mining	650 screened groups of patients	China
15	Artificial intelligence in critical illness and its impact on patient care: a comprehensive review.	2023	Saqib M, Iftikhar M, Neha F, Karishma F, Mumtaz H.	A comprehensive review		
16	The Aspects of Running Artificial Intelligence in Emergency Care; a Scoping Review.	2023	Masoumian Hosseini M, Masoumian Hosseini ST, Qayumi K, Ahmady S, Koohestani HR.	A Scoping Review		
17	The Impact and Issues of Artificial Intelligence in Nursing Science and Healthcare Settings	2023	Pailaha AD.	A review		
18	Artificial Intelligence in Emergency Medicine: Surmountable Barriers With Revolutionary Potential.	2020	Grant K, McParland A, Mehta S, Ackery AD	A review		
19	Artificial intelligence: opportunities and implications for the health workforce	2020	Hazarika I	A review		
20	AI in Health: State of the Art, Challenges, and Future Directions	2019	Wang F, Preininger A	A review		

### Quality evaluation of the selected articles

Given the time-sensitive nature of this scoping review, we opted for a structured yet streamlined approach to assess the quality of the selected articles. While the Critical Appraisal Skills Programme tool was initially considered, time constraints necessitated a more focused assessment. We prioritized key quality indicators, including study design, sample size, and relevance to our research questions, to identify the strengths and limitations of each article.

This pragmatic approach allowed for efficient evaluation and informed the synthesis of findings within this rapidly evolving field.

### Findings

The comprehensive search of the selected databases resulted in the identification of 20 articles that met the inclusion criteria. AI applications in emergency nursing encompass a wide range of areas, including triage, patient monitoring, diagnosis, treatment planning, and decision support systems. There are several potential applications of AI in emergency nursing. These applications include using AI algorithms to analyze patient data and predict adverse events or deterioration in the emergency department. Additionally, AI can be used to assist in triage decision-making, improving the accuracy and efficiency of patient prioritization.

### Triage

Machine learning algorithms were predominantly used to assess the severity of patient conditions to prioritize care delivery (8). The findings suggested that AI could potentially reduce wait times and improve patient outcomes (8). These results indicate the potential for AI to revolutionize the triage process in emergency nursing, leading to more efficient and accurate patient prioritization and ultimately improving patient outcomes (9).

Utilizing AI-powered triage systems to improve the accuracy and efficiency of patient prioritization in the emergency department. Furthermore, AI can assist in the identification of high-risk patients who may require immediate intervention, allowing healthcare providers to allocate resources accordingly (9).

### Patient monitoring

A significant number of studies focused on the application of AI in patient monitoring within the emergency department. These studies highlighted the use of AI-powered tools for continuous analysis of patient data and vital signs to detect concerning changes in a patient’s condition (10, 11). The findings indicated that AI-enabled patient monitoring could lead to early identification of deteriorating patient conditions, allowing for timely interventions and improved patient outcomes (12–14). AI applications in patient monitoring have the potential to enhance the accuracy and timeliness of detecting changes in a patient’s condition in the emergency department (13). AI can provide real-time monitoring and analysis of patient data, allowing healthcare providers to quickly identify deteriorating conditions and intervene promptly (13, 15). Additionally, AI can assist in the prediction of adverse events or complications in emergency nursing. Through its ability to analyze large amounts of patient data and detect patterns and trends, AI can aid in predicting adverse events and complications in emergency nursing (13, 15, 16).

### Diagnosis

AI’s role in diagnosis was examined in various studies, with a focus on the use of AI for the early detection of conditions. These studies indicate that AI can enhance diagnostic accuracy and speed, thereby facilitating swifter intervention and treatment (10, 15, 17–21). By implementing AI algorithms in the emergency nursing setting, healthcare providers can potentially detect diseases earlier and initiate appropriate treatment more quickly (18). AI has the potential to enhance diagnostic accuracy and speed in emergency nursing by facilitating earlier detection of conditions and enabling swift intervention and treatment (18–20).

### Treatment planning and decision support

AI’s application in treatment planning and decision support was highlighted in several studies. The use of AI algorithms to synthesize patient information and medical evidence was found to support clinical decision-making, leading to personalized care plans and potentially better patient outcomes (10, 15, 22). By harnessing the power of AI, emergency nursing practitioners can receive real-time, data-driven treatment recommendations that are tailored to each patient’s unique needs (15). This can result in more effective and efficient treatment planning, ultimately improving patient outcomes in the emergency department (15, 22). Integrating AI technologies, such as natural language processing, to enhance communication and documentation processes for nurses in the emergency department (15). Implementing AI-powered decision support systems to assist nurses in making clinical decisions and treatment plans. Based on the scoping review, the future of AI for emergency nursing at the emergency department holds great potential (15).

### Synthesis of AI technology in emergency nursing

Overall, the synthesis of the data extracted from the studies shows that AI technologies are employed in emergency nursing for various applications, from routine patient observations to complex decision-making scenarios. The integration of AI into emergency nursing workflows showed promising results regarding efficiency and patient care quality (10). These studies emphasize the potential benefits of AI in emergency nursing, including improved patient outcomes, enhanced decision-making, and increased efficiency in administrative tasks (13).

However, it is important to note that the current research on AI in emergency nursing is still limited and more robust studies are needed to fully understand the potential benefits and limitations of AI technology in emergency nursing (23).

Findings from this review suggest that using AI-based interventions has a positive effect on pain recognition, pain prediction, and pain self-management; however, most reports are only pilot studies. More pilot studies with physiological pain measures are required before these approaches are ready for large clinical trial (23).

A study showed that the ECG recommendation can effectively predict whether patients presenting at ED triage will require an ECG, prompting subsequent analysis and decision-making in the ED (12). Another study indicated that a real-time AI predictive model is a promising method for predicting adverse outcomes in ED patients with hyperglycemic crises. Further studies recruiting more patients are warranted (17). Research has demonstrated that recommendations for electrocardiograms can accurately forecast the necessity for an ECG in patients at emergency department triage, thus aiding further evaluation and decision-making processes in the ED setting (17).

### Education and training

Leveraging AI in emergency nursing education and training to enhance the knowledge and skills of nurses in handling emergency situations (24). These findings suggest that AI has great potential to revolutionize emergency nursing by improving patient outcomes, enhancing workflow efficiency, and supporting clinical decision-making (24). The future of AI in emergency nursing at the emergency department looks promising. However, there are several limitations and challenges associated with the implementation of AI in emergency nursing (25). These include the need for robust and validated AI algorithms, ensuring privacy and data security, addressing potential biases in AI systems, and providing adequate training and education to healthcare professionals for effective use of AI technologies (25). In summary, the future of AI in emergency nursing at the emergency department holds great potential for improving patient care and workflow efficiency.

### Challenges and ethical considerations

Despite the advantages, the studies also pointed out several challenges related to the integration of AI into emergency nursing, including the need for robust data infrastructure, concerns about patient privacy and data security, and the requirement for ongoing training and education for healthcare professionals to effectively use AI technologies (10, 14, 25, 26). Furthermore, there are ethical considerations that must be addressed when implementing AI in emergency nursing (27). These include ensuring transparency and explainability of AI algorithms, addressing biases and potential discrimination in AI systems, maintaining patient autonomy and consent, and avoiding overreliance on AI without human oversight (25, 26). In conclusion, while the future of AI in emergency nursing at the emergency department shows great promise, there are still various challenges and ethical considerations that need to be carefully addressed to ensure responsible and effective implementation.

## Discussion

This scoping review examined the emerging role of artificial intelligence in emergency nursing, revealing a field brimming with potential yet facing significant hurdles. Our analysis of 20 studies meeting the inclusion criteria highlighted a diverse range of AI applications, spanning triage optimization, patient monitoring, diagnostic assistance, treatment planning, and decision support systems.

The findings showed that in the area of triage, AI’s ability to analyze and interpret vast datasets swiftly can aid in the development of more accurate triage systems. Such systems have the potential to prioritize patient care delivery more effectively, allowing for rapid attention to the most urgent cases and potentially reducing waiting times. AI can also support the identification of subtle patterns that may not be immediately evident to human clinicians, contributing to better-informed triage decisions (23).

For patient monitoring, AI offers dynamic and real-time analytics that can predict clinical deterioration earlier than conventional monitoring systems (11, 14). These predictive alerts can equip nursing staff with crucial lead time to intervene preemptively and possibly mitigate the severity of patient outcomes (27). The continuous monitoring capabilities of AI can also alleviate some of the manual workload on nurses, allowing them to allocate their expertise where it is most needed.

Diagnosis is another area where AI can have a profound impact. With the development of advanced algorithms capable of diagnostic support, there are potential gains in the accuracy and speed of identifying medical conditions. Consequently, this could lead to more prompt initiation of appropriate treatments and better patient recovery trajectories (3).

The findings showed that treatment planning and decision support systems benefit from AI by providing evidence-based recommendations that are tailored to individual patient data. This individualization of care plans is crucial in emergency nursing, where every patient’s conditions and needs can vary greatly. AI’s computational power enables the handling of complex algorithms that take into account a multitude of variables, thus aiding nurses in crafting precise and effective treatment strategies (23). However, the application of AI in emergency nursing also presents significant challenges and considerations. There is a pressing need to ensure that nurses are adequately trained to employ these new tools and that they understand both the capabilities and limitations of AI technology (28). The integration of AI should not replace clinical judgment but rather serve as a supplementary aid that enhances the nurse’s ability to care for patients (3).

Moreover, the ethical concerns regarding patient privacy, data security, and the potential for algorithmic bias must be diligently addressed. The development of AI systems for healthcare requires a transparent approach, where the processes and reasoning of AI algorithms are accessible and comprehensible to healthcare professionals (28–30). This transparency is vital for building trust in AI applications and ensuring they align with the ethical standards of medical care (31).

The integration of artificial intelligence in emergency nursing, while promising, presents significant ethical challenges that warrant in-depth research. One pressing concern is the potential for algorithmic bias, where AI systems may perpetuate existing healthcare disparities by learning from biased datasets. Future research should focus on developing robust methods for detecting and mitigating bias in AI algorithms used in triage, diagnosis, and treatment recommendations. This includes investigating techniques like comparative performance analysis across diverse patient demographics, incorporating fairness constraints into algorithm development, and creating explainable AI tools that make bias detection transparent for healthcare professionals.

Another crucial area for future research is safeguarding patient privacy and data security in the age of AI-driven healthcare. As AI systems rely heavily on vast amounts of patient data, ensuring the confidentiality and integrity of this information is paramount. Research should explore privacy-preserving techniques like federated learning, which allows AI models to be trained on decentralized datasets without sharing sensitive patient information. Additionally, investigating the application of differential privacy mechanisms, which add noise to datasets to protect individual privacy while preserving data utility, could be beneficial in the context of AI-driven emergency nursing tools. Developing secure and privacy-preserving infrastructure for storing, accessing, and processing patient data used in AI applications is also crucial.

### Implications for emergency nursing practice

Emergency nurses can utilize AI to enhance triage accuracy and efficiency. By analyzing patient data such as vital signs, medical history, and presenting symptoms, AI algorithms can assist nurses in rapidly identifying high-risk patients and prioritizing care, leading to more effective triage decisions and potentially reducing wait times for critical cases. Furthermore, AI-powered monitoring systems can revolutionize patient care in the emergency department. These systems can continuously analyze patient data, detecting subtle changes and early warning signs of deterioration that may not be immediately apparent to human observation. This allows for proactive interventions, potentially preventing adverse events and improving patient outcomes. AI can also support nurses in making faster and more accurate diagnoses by analyzing medical images, EKGs, and other diagnostic data. This can expedite treatment decisions and improve patient care in time-sensitive situations. However, the successful integration of AI in emergency nursing requires a shift in practice. Nurses will need ongoing education and training to adapt to these new technologies and develop the skills to effectively utilize AI tools. Collaboration between nurses, physicians, data scientists, and AI specialists will be crucial to ensure responsible and ethical implementation. As AI takes on more routine tasks, nurses’ roles may evolve to focus on more complex decision-making, patient education, and providing compassionate, human-centered care.

### Implications for emergency nursing research

The emergence of AI in emergency nursing also sets a new direction for research:

1. Effectiveness Studies: Longitudinal studies assessing the effectiveness of AI tools in various emergency nursing scenarios are needed.
2. Ethical Research: with the rise of AI, there is a significant need for research into the ethical implications of its use, such as patient privacy and algorithmic biases.
3. Interdisciplinary Research: encouraging interdisciplinary research incorporating computer science, nursing, and other healthcare domains to foster the development of more nuanced AI tools tailored for emergency care.
4. Outcomes Research: Investigating the impacts of AI on patient outcomes and satisfaction levels is crucial for validating its role in clinical settings.

Overall, the adoption of AI in emergency nursing has far-reaching implications, necessitating a proactive approach to both practice and research to harness its potential responsibly and maximize benefits for patient care.

## Conclusion

The diverse applications of AI in emergency nursing, ranging from improved diagnostics to more efficient workflow management, show great potential for transforming the field. The application of AI in emergency nursing holds remarkable potential for reshaping emergency medicine. If leveraged responsibly, AI can aid in refining the precision and efficiency of emergency care, offer novel insights into patient health, and ensure that healthcare systems are better equipped to meet the demands of acute care. Nonetheless, realizing this potential requires a concerted effort to tackle the associated technical, ethical, and professional challenges.

By acknowledging these findings and fostering a culture of ethical AI use, emergency nursing can advance toward a future where technology-enabled care is the norm, not the exception. The need for ongoing discourse among clinicians, technologists, policymakers, and patients is crucial to ensure that AI in emergency nursing is implemented responsibly and ethically.

## Limitation

Our scoping review is constrained by certain limitations. The range and diversity of AI applications in emergency nursing are vast, yet current research may not be comprehensive or exhaustive. Studies may vary in methodology, scope, and context, which could impact the generalizability of our findings. Furthermore, the rapid evolution of AI technology means that our review might not reflect the very latest advancements. Finally, despite the potential of AI to transform emergency nursing, broader system-wide evaluations are required to fully understand its implications, including areas such as data security and ethical considerations in implementation.
